# Bioinformatics-based identification of SPNS3 (Spinster homolog 3) as a prognostic biomarker of apoptosis resistance in acute myeloid leukemia

**DOI:** 10.1080/21655979.2021.1982303

**Published:** 2021-10-05

**Authors:** Yang Hong, Xiaopeng Tian, Mengmiao Wang, Cheng Chen, Aining Sun

**Affiliations:** a Department of Hematology, Jiangsu Institute of Hematology, the First Affiliated Hospital of Soochow University, National Clinical Research Center for Hematologic Diseases, Suzhou, China; b Institute of Blood and Marrow Transplantation, Medical College of Soochow University, Jiangsu Institute of Hematology, the First Affiliated Hospital of Soochow University, Collaborative Innovation Center of Hematology, National Clinical Research Center for Hematologic Diseases, Soochow University, Suzhou, China

**Keywords:** SPNS3, FLT3-ITD, ceRNA, apoptosis, acute myeloid leukemia

## Abstract

Spinster homolog 3 (SPNS3) belongs to the Spinster (SPNS) family which participates in sphingolipid transportation through the cell membrane. However, the functions of SPNS3 in acute myeloid leukemia (AML) are unknown. This study obtained SPNS3 from a gene set that was related to AML relapse and evaluate whether high SPNS3 expression induced apoptosis resistance in an AML cell line, which is consistent with the role of SPNS3 as a marker of poor prognosis in the clinic. Moreover, internal tandem duplication of FMS-like tyrosine kinase 3 (FLT3-ITD) mutation and the AC127521.1/ MIR-139/SPNS3 competing endogenous RNA axis were found to regulate SPNS3 expression. In addition, we noted that SPNS3 may play an important role in the Sphingosine-1-phosphate signal pathway that is involved in the maintenance of the AML microenvironment. These results highlight the anti-apoptosis effect of SPNS3 in AML, and the potential mechanism mediating this effect was explored through bioinformatics.

**Abbreviations:** AML: acute myeloid leukemia; FLT3-ITD: internal tandem duplication of FMS-like tyrosine kinase 3; SPNS3: spinster homolog 3; SPNS1: spinster homolog 1; SPNS2: spinster homolog 2; GO: gene ontology; S1P: sphingosine-1-phosphate; ceRNA: competing endogenous RNA; dAML: acute myeloid leukemia at diagnosis; iAML: acute myeloid leukemia after induction chemotherapy; rAML: acute myeloid leukemia at relapse; DEGs: differentially expressed genes; BP: biological processes; CC: cellular components; MF: molecular functions; MRD: minimal residual disease; EFS: event-free survival; OS: overall survival; KEGG: Kyoto Encyclopedia of Genes and Genomes; SPHK: Sphingosine kinase.

## Introduction

Acute myeloid leukemia (AML) is a common hematologic malignancy that occurs from the abnormal clonal expansion of myeloid blasts and gradually inhibits normal hematopoiesis [1]. AML relapse after chemotherapy is a major problem in the field of AML treatment [2]. Internal tandem duplication of FMS-like tyrosine kinase 3 (FLT3-ITD), which has been a focus area of active research, is a proven relapse marker in AML [3]. However, the mechanism involved in FLT3-ITD mutation and the relative transcriptome variance is largely uninvestigated.

Spinster homolog 3 (SPNS3) is a gene that belongs to the Spinster superfamily. Annotations in gene ontology (GO) that involve SPNS3 include transmembrane transporter and sphingolipid transporter activities. Nonetheless, the specific function of SPNS3 is rarely known, whereas the functions of other members of the Spinster superfamily, namely SPNS1 and SPNS2, have been widely investigated. SPNS1 mainly participates in autophagic cell death [4], and SPNS2 often acts as an S1P transporter [5]. Furthermore, in species ranging from elephant sharks to mammals, both SPNS2 and SPNS3 have tandem locations in the genome [6]. Although the function of SPNS3 has not yet been elucidated, the high degree of conservation of SPNS3 indicates its importance in biological functions [7].

We hypothesized that SPNS3 may be essential for the biological behavior of the AML cells and aimed to investigate the role and regulatory mechanism of SPNS3 in AML. Primarily, we planned to definite whether SPNS3 could serves as a biomarker that indicates the prognosis of AML at the first diagnosis and build the correlation of the AML cells’ phenotype with the clinical significance of SPNS3 through experiments. Secondarily, bioinformatics-based analysis was expected to reveal the regulatory factors of SPNS3 expression for designing targeting therapy in the future.

## Methods and materials

### Acquisition of study datasets

Four datasets (GSE75086, GSE14468, GSE17855, and GSE52891) were acquired from the Gene Expression Omnibus (GEO) database (https://www.ncbi.nlm.nih.gov/geo, National Center for Biotechnology Information Search database) for the data analysis in this study. Furthermore, the RNA-seq data of 187 AML samples were obtained from the TARGET website (https://ocg.cancer.gov/programs/target, TARGET-AML program). After excluding 74 samples that could not be matched with relevant clinical data, a total of 113 AML samples were included in this study. All of the data used in the analyses are freely available online in the public domain.

### GEO2R analysis

The online analysis tool GEO2R (https://www.ncbi.nlm.nih.gov/geo/geo2r/, National Center for Biotechnology Information Search database) was utilized to select the differentially expressed genes (DEGs) in the GEO datasets that were analyzed in our study. Next, we derived the adjusted ***P***-value and |log_2_FC| of the DEGs, and an adjusted ***P*** < 0.05 and |log_2_FC|≥1.0 were set as the cutoff criteria.

### WGCNA analysis with a TARGET-AML Dataset

To demonstrate the important role of SPNS3 in AML, we used the R Package WGCNA [8] to build the co-expression network of the relapse-related genes, including SPNS3, and established a clinically significant correlation with SPNS3 expression.

### General analysis of SPNS3 on the gene expression profiling interactive analysis website

Gene Expression Profiling Interactive Analysis (GEPIA; http://gepia2.cancer-pku.cn) [9], an open-access online RNA-seq analysis tool, was used to analyze the pan-cancer SPNS3 expression and differences in the SPNS3 expression between AML and normal samples in The Cancer Genome Atlas (TCGA) database. Next, by using the Kaplan-Meier survival analysis with a 50% (median) cutoff for both low- and high- expression groups, we evaluated the overall survival (OS) of the patients in whom the SPNS3 gene was targeted. Moreover, to infer the regulation and functions of SPNS3, we performed a correlation analysis between SPNS3 and its related genes using Pearson’s correlation analysis.

### Survival analysis in prognoscan and the TARGET-AML database

To validate the prognostic significance of SPNS3 expression in AML, we used the PrognoScan [10] website to analyze OS based on the variations in the expression of SPNS3 (using the Kaplan-Meier method plus the log-rank test) in the GSE12417 dataset; the TARGET-AML database was similarly analyzed on both OS and event-free survival (EFS) using ”lifelines” Package in Python v3.8 [11].

### Selecting differentially expressed mRNA, miRNAs, lncRNAs based on SPNS3 expression

We used R language 3.8 version edgeR package [12] to undertake the comparison of mRNA, miRNA, and lncRNA expressions between the high SPNS3 and low SPNS3 groups after normalizing the expression profile of all genes (average value >1). We defined the screening criteria as a false discover rate (FDR) < 0.05 and |log_2_FC| >2.

### Gene enrichment analysis of DEGs

To investigate the potential function of SPNS3, we uploaded the abovementioned differentially expressed mRNAs to the Database for Annotation, Visualization, and Integrated Discovery (DAVID) tools [13,14]and conducted a website-guided gene enrichment analysis. We selected GO and Kyoto Encyclopedia of Genes and Genomes (KEGG) pathway results to annotate the role of SPNS3 expression. To screen the results, we defined an FDR <0.05 as statistically significant.

### Gene Set Enrichment Analysis (GSEA)

Simultaneously, we selected RNA-seq data samples from the SPNS3 high-expression group in TARGET-AML and analyzed the samples using the online tool available on the GSEA website [15,16].

### Competing endogenous RNA prediction and validation

The TargetScan database is a well-known website for predicting miRNAs targets [17].Using TargetScan, we predicted the miRNAs that potentially target SPNS3. Furthermore, we predicted the lncRNA-miRNA relationship based on the LncBase Predicted v.2 [18]. Moreover, we merged both the predicted results with the sorted DEGs that were powered by edgeR and validated the merged lncRNA-miRNA-mRNA competing endogenous RNA (ceRNA) relationship using the TARGET RNA-seq data.

### Cell cultures

MOLM-13 (FLT3-ITD-positive AML cells) and HEK-293 T cells were purchased from the American Tissue and Cell Culture cell-line bank. MOLM-13 were cultured in Roswell Park Memorial Institute (RPMI) 1640 medium (Gibco) with 10% FBS (Biological Industries, BI) and 100 μg/mL streptomycin and 100 IU/mL penicillin (Beyotime Biotechnology) at 37°C in a humified atmosphere containing 5% CO_2_. HEK-293 T cells were cultured in Dulbecco’s Modified Eagle’s medium (DMEM; Gibco) with 10% FBS (BI) at 37°C with 5% CO_2_.

### Plasmid construction and transfection in MOLM-13

SPNS3 knockdown in MOLM-13 was performed to validate the correlation between SPNS3 expression and cell apoptosis in AML. Three pairs of 58-bp oligonucleotides (shRNA1, shRNA2, and shRNA3) that encoded a 21-bp shRNA were designed to silence the human SPNS3 gene in MOLM-13. In addition, one pair of scrambled shRNA was used as a control. The shRNA sequences that targeted human SPNS3 are shown in Table S2, with interfering sequences acquired from the website of Sigma Company (www.sigmaaldrich.com). The expressed sequence tags (EST) were analyzed using the basic local alignment search tool from the National Center for Biotechnology Information (NCBI) to verify that the shRNA only targeted human SPNS3. Three pairs of shRNA-encoded nucleotides were designed to target various parts of SPNS3 mRNA and were synthesized by GENEWIZ, Inc. Equal volumes of sense and antisense oligonucleotides were mixed and heated at 94°C for 4 min, then cooled in 5-min intervals at 70°C, 60°C, 50°C, 40°C, 30°C and 20°C. The annealed oligonucleotides were inserted into a PLKO.1-GFP vector using T4 DNA ligase (New England Biolabs, MA, USA), and the PLKO.1-GFP vector was digested with EcoRI and AgeI (New England Biolabs, MA, USA). Recombinant vectors were validated by sequencing (GENEWIZ, NJ, USA). The envelope plasmid (pMD2.G) and packaging plasmid (psPAX2) were co-transfected with recombinant plasmid into HEK-293 T cells for lentivirus production.

Lentiviruses from HEK-293 T cell supernatants were harvested 48 h after transfection. Activated MOLM-13 was transduced with packaged lentiviruses (scrambled, hSPNS3sh1, hSPNS3sh2 and hSPNS3sh3) for 12 h, followed by a change to regular complete medium culture for the next 72 h. Then, we tested the infectious efficiency based on the green fluorescent protein (GFP) expression that was detected by flow cytometry.

### Quantitative real-time PCR

The transfected cells were harvested in Eppendorf tubes and lysed using TRIzol (Invitrogen, CA, USA) following the manufacturer’s protocol. Then, 1 μg total cellular RNA was reverse-transcribed to cDNA with Reverse Transcriptase M-MLV (RNase H-; TaKaRa, Japan) to a final volume of 10 μL, followed by the addition of 1 μL cDNA into TB Green® Premix Ex Taq™ (Tli RNaseH Plus; TaKaRa, Japan) to obtain a final volume of 20 μL. Quantitative real-time PCR was performed using the ABI QuantStudio3 Detection System (Applied Biosystems, Carlsbad, CA, USA). The relative expression of the samples was measured using the ΔΔ^CT^ method, and each sample was run in triplicate. The housekeeping gene GAPDH was used to normalize individual samples. A list of primer sequences is provided in Table S1.

### Annexin V-FITC/7-AAD flow detection

The transfected cells were harvested and washed twice with Annexin-V binding buffer. After discarding the supernatant, 5 μL APC-Annexin-V and 5 μL 7-AAD were separately added to the resuspended cells. Then, the resuspended cells were incubated in the dark for 20 min, at room temperature. In the end, we washed the samples with binding buffer again and transferred them to the flow tube for flow cytometry.

### Statistical analysis

The data that were included for analysis and the progress of analysis in this study were checked by two people. All laboratory experiments in this study were repeated at least 3 times, and similar results were obtained. The Student’s *t*-test was used for numerical comparisons and the chi-square test was used for the intergroup comparison of categorical data. The results of the flow cytometry experiments were analyzed in FlowJo v10. The statistical graphs were drawn in Prism Graphpad v8.3 software or were supported by the ”seaborn” and ”pyecharts” packages coded in Python programming v3.8. Data are presented as mean ± standard deviation (SD). ***P***-value of < 0.05 was considered to be statistically significant and significance is presented as *P < 0.05, **P < 0.01, or ***P < 0.001.

## RESULTS

This study focused on a barely investigated correlation between SPNS3 and AML relapse. We believed that the significantly high expression of SPNS3 in AML may be essential for the biological function of AML cells and explored the underlying mechanisms. Our research generated evidence of SPNS3-induced apoptosis resistance in AML which partially explained the poor survival of AML patients with relatively high SPNS3 expression. Using bioinformatics, the relationships between SPNS3 and FLT3-ITD mutation or the AC127521.1/MIR-139/SPNS3 ceRNA axis were identified.

### Identification of DEGs related to AML relapse

First, we divided the gene expression dataset (GSE75086) samples into three groups: AML at diagnosis (dAML), AML after induction chemotherapy (iAML) and AML at relapse (rAML) according to the sample description. After the GEO2R analysis between iAML and rAML, a total of 511 DEGs (FDR<0.05, |log_2_FC|>1) related to relapse were identified (Figure S1A). Furthermore, we utilized the WGCNA package, together with TARGET RNA-seq data and corresponding clinical data to construct the co-expression network based on the DEGs (Figure S1B-D). Finally, we found that high SPNS3 expression positively correlated with AML relapse (***P*** = 0.043), death (***P*** = 0.023), FLT3-ITD mutation (***P*** < 0.000), WT1 mutation (***P*** = 0.030), and minimal residual disease (MRD) at end of course 2 (***P*** < 0.000) and negatively correlated with EFS (***P*** = 0.019), t(8;21) chromosomal abnormality (***P*** = 0.001), cytogenetic complexity (***P*** = 0.046), complete remission (CR) at end of course 2 (***P*** < 0.000) (Table 1).

**Table 1.** The correlation between SPNS3 expression and clinical phenotypes in the WGCNA co-expression network

	Correlation	*P*-value
Relapse*	0.19	0.043
EFS*	−0.22	0.019
Death*	0.21	0.023
t(8;21) **	−0.29	0.001
Cytogenetic complexity*	−0.19	0.046
FLT3-ITD***	0.55	0.000
WT1 mutation*	0.20	0.030
MRD at the end of Course 2***	0.35	0.000
CR at the end of Course 2***	−0.50	0.000

*: *P* < 0.05; **: *P* < 0.01; ***: *P* < 0.001.

CR: complete response; FLT3-ITD: internal tandem duplication of FMS-like tyrosine kinase 3; EFS: event-free survival; MRD: minimal residual disease.

### Characteristics of SPNS3 expression and correlation with overall survival in AML

**Figure 1.**
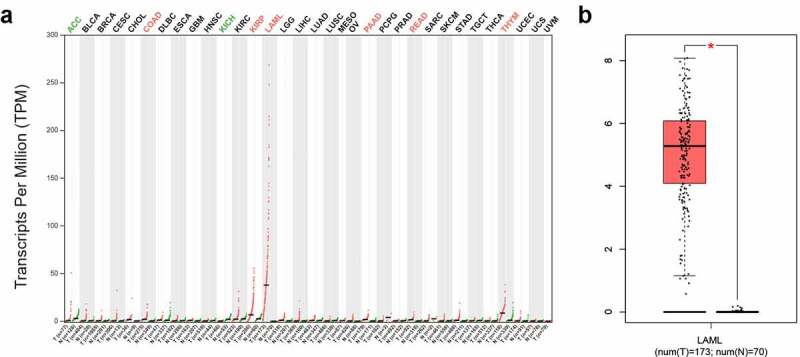
SPNS3 high expressed in acute myeloid leukemia (AML). a. Pan-cancer analysis showed that the average SPNS3 expression was highest in AML; b. Compared to normal tissues, SPNS3 expression significantly increased in AML

**Figure 2.**
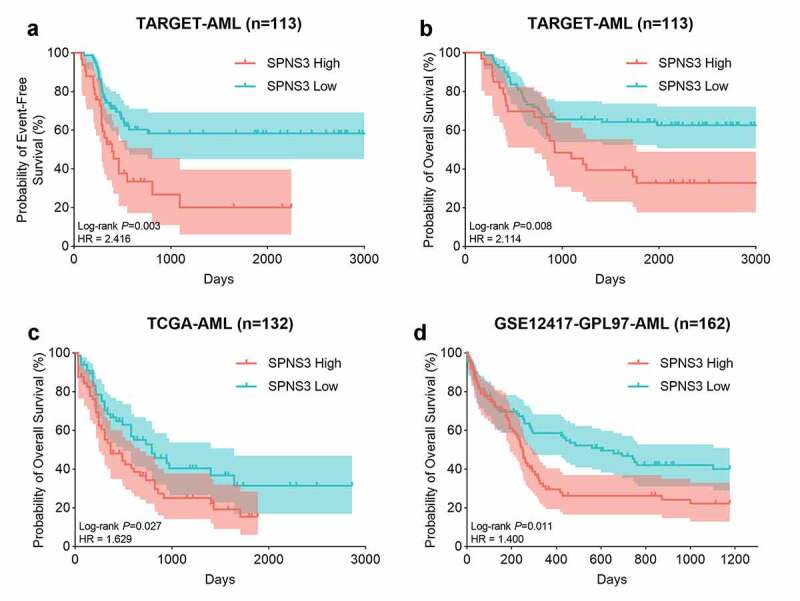
High SPNS3 expression significantly influenced the survival of AML patients. a, b. The Kaplan–Meier survival analyses to both the event-free survival and the overall survival of the AML patients for the SPNS3 expression in the TARGET database. c, d. The overall survival of the patients in the TCGA database and the GSE12417 dataset acquired from the prognoscan website analyzed by the Kaplan-Meier method to verify the effect of SPNS3 to the survival of AML patients

Using GEPIA, we further elucidated the expression of SPNS3 in 38 different types of neoplasms and found that average SPNS3 expression was highest in AML (Figure 1(a)) and was significantly enhanced compared to the expression in the normal tissue samples (***P*** < 0.05) (Figure 1(b)) which suggested that SPNS3 expression might play an important role in the onset and development of AML. To evaluate whether SPNS3 expression correlates with the OS of AML patients, we performed a survival analysis of AML with variable SPNS3 expression in three independent databases. Meanwhile, the EFS of the AML patients in the TARGET database was evaluated to demonstrate the effect of SPNS3 to the progression of AML. In general, SPNS3 expression significantly influenced both EFS (***P*** = 0.003, hazard ratio (HR) = 2.416; Figure 2(a)) and OS (P = 0.008, HR = 2.114; Figure 2(b)) of AML in the TARGET database and OS in TCGA database (***P*** = 0.027, HR = 1.629; Figure 2(c)), and in PrognoScan database (***P*** = 0.011, HR = 1.400) (Figure 2(d)).

### Functional enrichment analyses of DEGs related to SPNS3 expression

**Figure 3.**
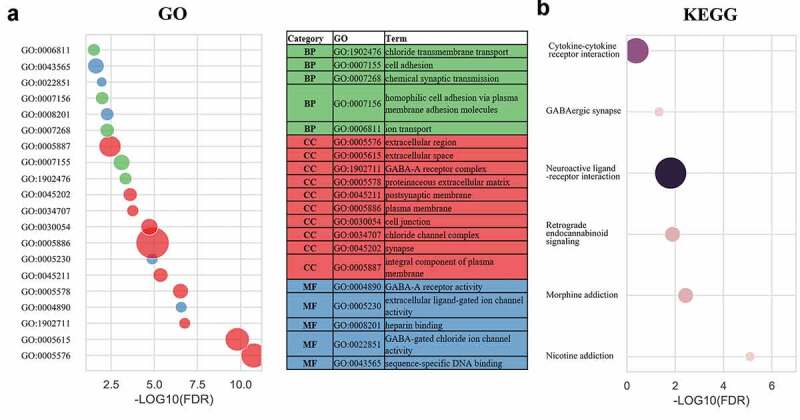
Gene enrichment analysis indicated a potential mechanism that correlated with high SPNS3 expression. Significantly (false discovery rate (FDR)<0.05) enriched GO terms (a) and KEGG pathways (b) of differentially expressed genes (DEGs) between the SPNS3-high and -low groups in the TARGET database

To investigate the role of SPNS3 in AML, we divided 113 samples into the SPNS3-high (n = 57) and SPNS3-low group (n = 56) groups according to the levels of SPNS3 expression recorded in the TARGET database and used the edgeR package to analyze the intergroup difference in mRNA expression. Accordingly, we identified 107 up-regulated DEGs and 608 down-regulated DEGs (FDR<0.05, |log_2_FC|>2; Figure S2A, B). Based on the gene enrichment analysis performed with data from the DAVID database (FDR<0.05; Figure 3(a,b)), the GO enrichment terms were classified into cellular components (CC), biological processes (BP), and molecular functions (MF). The results of GO enrichment indicated that differentially expressed mRNA were mainly enriched in the CC block, including the extracellular region, extracellular space, GABA-A receptor complex, proteinaceous extracellular matrix, postsynaptic membrane, plasma membrane, cell junction, chloride channel complex, synapse, and integral component of the plasma membrane. The MF block showed that the DEGs were significantly enriched for GABA-A receptor activity, extracellular ligand-gated ion-channel activity, heparin-binding, GABA-gated chloride ion-channel activity and sequence-specific DNA binding. For the BP block, the DEGs were enriched for chloride transmembrane transport, cell adhesion, chemical synaptic transmission, homophilic cell adhesion via plasma membrane-adhesion molecules, and ion transport. Furthermore, the results of KEGG pathway analysis showed that DEGs were mainly enriched in the pathways related to nicotine addiction, morphine addiction, retrograde endocannabinoid signaling, neuroactive ligand-receptor interaction, GABAergic synapse and cytokine-cytokine receptor interaction.

### The role of the ceRNA axis in SPNS3 expression

**Figure 4.**
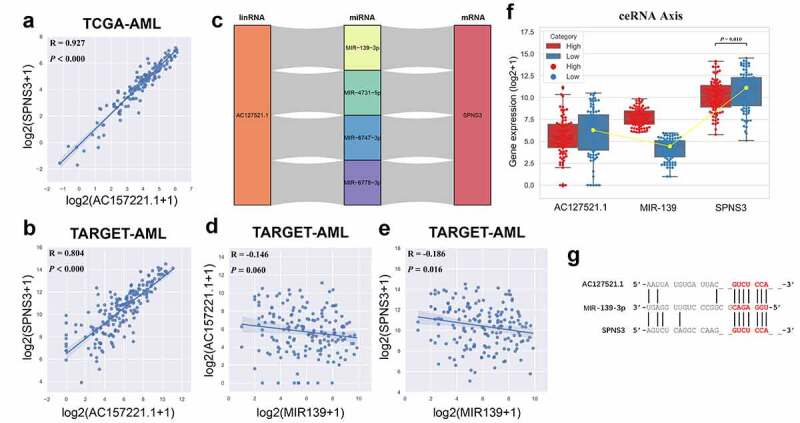
Identification of the ceRNA-axis regulation of SPNS3 expression. a, b. LncRNA AC127521.1 expression positively correlated with SPNS3 expression in AML samples from both the TCGA and the TARGET databases. c. Four potential lncRNA-miRNA-mRNA-ceRNA axes were predicted by merging the result from the TargetScan and DIANA databases. d, e. MIR-139 expression negatively correlated with both LncRNA AC127521.1 and SPNS3 expression in AML samples in the TARGET database. f. By comparing the AML samples of the SPNS3-high and SPNS3-low groups in the TARGET database, the AC127521.1/MIR-139/SPNS3 ceRNA axis was validated in terms of the RNA expression level, and the ceRNA relationship was more apparent in the SPNS3-high group. g. The alignment of the MIR-139 sequence with predicted binding sites in the regions of LncRNA AC127521.1 and mRNA SPNS3 is shown

The abovementioned gene enrichment results elucidated how high SPNS3 expression influences the biological functions in AML. However, the factors regulating SPNS3 expression needed to be elucidated. Thus, we found the anti-sense lncRNA AC127521.1 expression positively correlated with SPNS3 mRNA expression, both in TCGA and TARGET databases (Figure 4(a,b)). Furthermore, four potential ceRNA relationships were predictively identified based on the TargetScan and DIANA databases (Figure 4(c)), and we validated these potential relationships in TARGET RNA-seq data. The results showed that MIR-139 expression negatively correlated with both lncRNA AC127521.1 and mRNA SPNS3 expression (Figure 4(d,e)). Taken together, the AC127521.1/MIR-139/SPNS3 axis was validated as constituting a trusted ceRNA relationship (Figure 4(f,g)).

### FLT3-ITD mutation and SPNS3 expression

**Figure 5.**
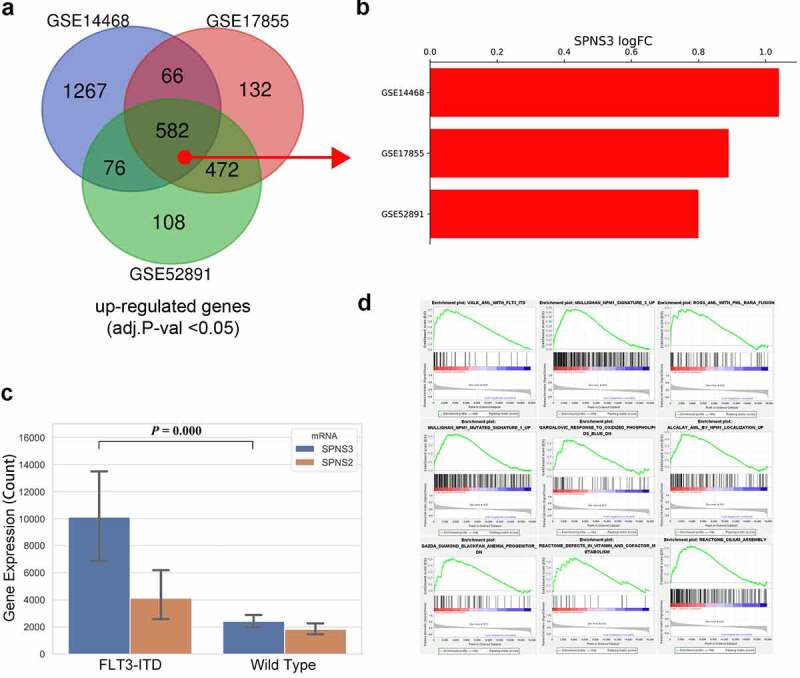
Identification of the FLT3-ITD mutation as an important regulator of SPNS3 expression. a. 582 upregulated genes (adj.***P***-val<0.05) in FLT3-ITD-positive AML were selected after the venn analysis of three GEO datasets (GSE14468, GSE17855, and GSE52891). b. Log2 value of the fold-change in SPNS3 expression indicated a consistently high expression of SPNS3 in FL3-ITD-positive AML in the above mentioned three datasets. c. Analysis of gene expression in the TARGET database showed that SPNS3 expression was significantly high in FLT3-ITD-positive AML samples whereas there was no apparent intergroup difference in SPNS2 expression. **D**. The AML samples with high SPNS3 expression in the TARGET database tended to be enriched with FLT3-ITD mutations on the GSEA

It is well accepted that genetic mutations are the main cause of cancer. As shown in the WGCNA co-expression analysis, the FLT3-ITD mutation significantly correlated with SPNS3 expression. An extensive analysis showed that the FLT3-ITD mutation may be the main cause of high SPNS3 expression. Furthermore, using the GEO2R tool to compare and analyze FLT3-ITD-positive and negative samples in three independent GEO datasets (GSE14468, GSE17855, and GSE52891) (Table 2), Venn analysis was performed to derive the intersection of the DEG profiles (Figure 5(a)). Intriguingly, SPNS3 was located in the intersection and had a consistently high expression in the FLT3-ITD group (Figure 5(b)). This difference between the high and low SPNS3 groups was validated in the TARGET database (Figure 5(c)). Furthermore, GSEA analysis revealed the AML samples with high SPNS3 expression tended to be enriched with the FLT3-ITD mutation (Figure 5(d)).

**Table 2.** Results of statistical analysis of the three microarray databases derived from the GEO database

Database ID	FLT3-ITD AML	Wild-Type AML	Total number
**GSE14468**	126	335	461
**GSE17855**	48	189	237
**GSE52891**	5	18	23

GEO, Gene Expression Omnibus; FLT3-ITD AML, AML with FLT3-ITD somatic mutation; Wild-Type AML, AML with wild-type FLT3 gene.

### SPNS3 knockdown induces apoptosis in MOLM-13 cells

**Figure 6.**
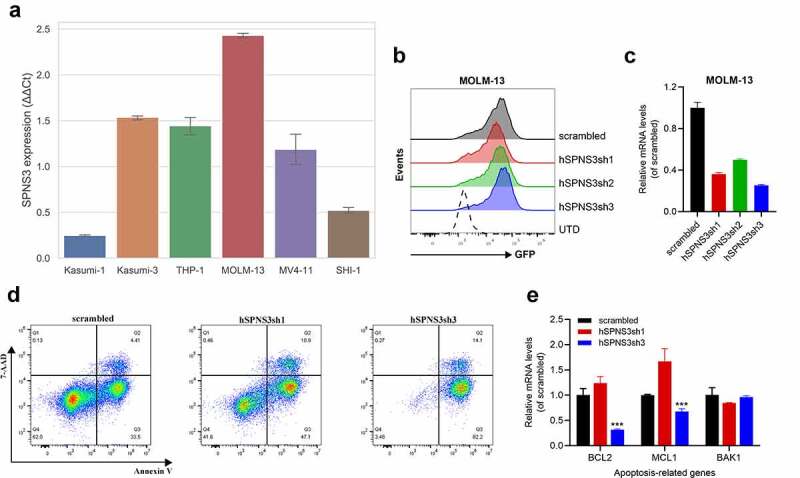
High SPNS3 expression induced apoptosis resistance in AML. a. qRT-PCR in six variant AML cell lines showed the highest SPNS3 expression in the FLT3-ITD+ cell line MOLM-13. b. The transfection efficiency was measured by using green fluorescent protein (GFP). The ratio of GFP+ cells of the four groups of the transfected MOLM-13, namely scrambled, hSPNS3sh1, hSPNS3sh2, and hSPNS3sh3, were similar and all higher than 90%. c. SPNS3 expression decreased in SPNS3 knocked-down MOLM-13 cells compared to the scrambled control. SPNS3 expression decreased more than 60% in hSPNS3sh1- and hSPNS3sh3- transfected MOLM-13 cells. d. Compared to the scrambled control, knocking down SPNS3 induced significant apoptosis in MOLM-13 cells. e. Relative quantitative expression of anti-apoptotic and pro-apoptotic genes

Firstly, we found that SPNS3 expression varied in six variants of AML cell-lines; therefore, we chose the cell-line with the highest SPNS3 expression (MOLM-13) for further experiments (Figure 6(a)) where MOLM-13 was transfected with SPNS3-targeting shRNAs for 12 h. The transfection efficiency of the four groups of the transfected MOLM-13 was ensured to be more than 90% GFP+ cells before undertaking the detection of cell apoptosis (Figure 6(b)). The SPNS3 expression decreased significantly in the MOLM-13 cells transfected with shRNA 1–3 (MOLM-13-hSPNS3sh1-3) compared to that in the MOLM-13 cells transfected with the scrambled sequence (MOLM-13-scrambled) (Figure 6(c)). 72 h after infection, the two groups of MOLM-13 cells with the highest efficiency of SPNS3 knocking down, namely MOLM-13-hSPNS3sh1 and MOLM-13-hSPNS3sh3 together with MOLM-13-scrambled underwent the further apoptosis detection through flow cytometry. As a result, we demonstrated that knocking down SPNS3 in MOLM-13 significantly increased the apoptosis of MOLM-13 cells (Figure 6(d)). In addition, the expression of anti-apoptosis genes, including B cell lymphoma 2 (BCL-2) and myeloid cell leukemia sequence 1 (MCL-1), decreased significantly in the most apparent SPNS3 knock-down group, namely MOLM-13-hSPNS3sh3, whereas there was no significant difference in the expression of the pro-apoptosis gene BAK1 (Figure 6(e)).

## Discussion

In this study, we demonstrated that high SPNS3 expression conferred an anti-apoptotic effect in AML and was responsible for the poor prognosis of AML patients. Furthermore, FLT3-ITD mutation and the AC127521.1/ MIR-139/SPNS3 ceRNA axis may be the drivers of high SPNS3 expression.

AML treatment has entered a new era of combination therapy with venetoclax and azacytidine, which have achieved a high percentage of clinical remissions in newly diagnosed patients [19]. However, upfront and acquired drug resistance partially indicate the clinical and genetic heterogeneity of AML, and there is an urgent need for the discovery of more effective potential therapeutic targets [20]. After sorting the alternative targeted genes that correlated with AML relapse, we focused on SPNS3, which has a significantly high expression in AML. In the current literatures, SPNS3 is recognized as an atypical solute carrier belonging to the major facilitator superfamily type [21] and participates in sphingolipid pathways to regulate airway hyperresponsiveness and mast cell activation in asthma patients [22]. To our best knowledge, our work is the first research into the pathological role of SPNS3 in AML. Encouragingly, our results indicate that SPNS3 expression is positively correlated with the resistance of AML cells to apoptosis and that SPNS3 knockdown enables a significant induction of apoptosis in the FLT3-ITD+ cell line MOLM-13 by inhibiting the expression of anti-apoptotic genes, namely BCL-2 and MCL-1. Together, the evidence indicates that SPNS3 may be a potential target for AML therapy and that SPNS3-targeted therapy and that SPNS3-targeted therapy might improve the effect of venetoclax in combination with azacytidine treatment.

To investigate the regulators of SPNS3 expression at the transcriptome level, we evaluated the genome recurrent-change FLT3-ITD mutation for SPNS3 expression. FLT3 is a proto-oncogene that is involved in key steps of hematopoiesis, such as proliferation, differentiation, and survival. Internal tandem duplication of FLT3 is the commonest form of mutation [23]. PI3K/AKT, MAPK/ERK, and STAT5 are the main signal transduction pathways that are activated by FLT3-ITD. Remarkably, an intracellular FLT3-ITD mutation indicated activation along with tyrosine phosphorylation of several signaling intermediates and anti-apoptotic signals that delay cell death [24]. Our study provided considerable insight the role of FLT3-ITD mutation for SPNS3 expression in AML. Thus, we found that the FLT3-ITD-positive groups showed a high expression of SPNS3 than the negative groups in all four of the recommended independent research studies. The FLT3-ITD mutation may drive the expression of SPNS3, and SPNS3 could become an alternative therapeutic target to help overcome the relatively poor prognosis of patients with FLT3-ITD-positive AML.

The ceRNAs are defined as transcripts that cross-regulate each other by competing for shared miRNAs [25]. In the human genome, more than 500 miRNA genes are estimated to function as miRNA response elements to mediate human mRNAs [26]. MIR-148a-3p and MIR-9, which are referred to in the latest reports of the effect of miRNAs in AML, play an important role in the etiopathogenesis of AML [27,28]. Furthermore, similar to these two miRNAs, the newly identified MIR-139 is involved in the regulation of the coding gene, SPNS3, and serves as the ceRNA that changes the AML cell phenotype. The accumulated evidence of the disarrangement of non-coding RNAs in AML provides a deep insight into the mechanism of AML as well as the fact that the landscape of the non-coding RNA network may generate new biomarkers and potential therapeutic targets for the treatment of AML patients. To explain the pathological significance of the high expression of SPNS3 in AML, we not only undertook GO, KEGG pathway gene enrichment and GSEA to show the main biological involvement, but also closely analyzed the role of SPNS3 in sphingosine-1-phospahte (S1P) signaling pathway and similar to other phosphosphingolipids, regulates complex cellular function in the tumor microenvironment where it plays a role as a signaling molecule that is involved in cell-cell communication [29]. S1P serves as an effective and biologically active signaling molecule, that can promote carcinogenesis by mediating cell proliferation, survival, migration, vascularization, etc [30]. In one form, S1P as the first messenger, undergoes extracellular release through autocrine or paracrine mechanisms [31], although the SPNS protein family (including SPNS1, SPNS2, and SPNS3) are responsible for the transport of S1P out of cells, which then combines with cell membrane receptors (S1PRs) and activates downstream signaling. Sphingosine kinase (SPHK) facilitates S1P and S1P diphosphatase (SPP) degradation maintains a balance between S1P generation and decomposition [32]. According to our findings, for the anti-apoptotic effect in AML, SPNS3 is likely to actively participate in the S1P signal pathway. Moreover, we found that sphingosine-1-phosphate (SGPP1), the gene encoding the expression of S1P diphosphatase 1 (SPP1), was significantly correlated negatively with SPNS3 expression, whereas the expression of SPHK2 was positively correlated with SPNS3 expression in both the TARGET and TCGA databases (Figure S3).

The results of this study enhance the understanding that SPNS3 promotes resistance against apoptosis, which accounts for the poor survival in AML to some extent, and high SPNS3 expression is a marker of poor prognosis in AML that is mediated through its anti-apoptotic effect. SPNS3 expression tends to be higher in FLT3-ITD-positive AML. Furthermore, we predicted that the AC127521.1/MIR-139/SPNS3 ceRNA axis is a validated pathway for regulating SPNS3 expression.

We are aware that our research may have at least two limitations. First, our results of the mechanism of SPNS3 expression and functions largely depended on bioinformatics and data interpretation, which need to be supported by additional research evidence. Second, although the prognosis value of SPNS3 was examined retrospectively in multiple independent datasets, it is necessary to verify the abovementioned results in a large, prospective study.

## Conclusion

Above all, we showed that SPNS3, a gene that is correlated with AML relapse, induces apoptosis resistance in AML and may be regulated by the FLT3-ITD mutation and the AC127521.1/MIR-139/SPNS3 ceRNA axis. Moreover, both the FLT3-ITD mutation and the AC127521.1/MIR-139/SPNS3 ceRNA axis were responsible for SPNS3 expression to enhance the S1P extracellular signal pathway while inhibiting apoptosis in AML cells. The prospect of identifying an important role of SPNS3 in AML, serves as a continuous incentive for future research.

## Supplementary Material

Supplemental Material

## Highlights

1. SPNS3 expression is significantly high in acute myeloid leukemia (AML) and serves as a survival indicator.

2. Silencing of SPNS3 leads to significant apoptosis of AML cells in vitro

3. SPNS3 may activate the S1P extracellular signal pathway to maintain biological behavior that is regulated by the FLT3-ITD mutation and the AC127521.1/ MIR-139/SPNS3 competing endogenous RNA axis.

## Acquisition of data and materials

All the clinical and gene expression data sets that were utilized and/or analyzed during the current study are available in publicly available databases.
