# Supplementary material for: Bioinformatics-based identification of SPNS3 (Spinster homolog 3) as a prognostic biomarker of apoptosis resistance in acute myeloid leukemia
Source: Bioengineered. 2021 Oct 5;12(1):7837–48. doi: 10.1080/21655979.2021.1982303 (PMC8806827; doi:10.1080/21655979.2021.1982303)
Supplement: Supplemental Material [file KBIE_A_1982303_SM6069.zip › supplementary/SUPPLEMENTAL FILES.docx]

**SUPPLEMENTAL FILES**

**Supplemental File 1: Figure S1. Establishment of the WGCNA co-expression network of DEGs related to AML relapse. A.** 511 DEGs (FDR<0.05, |logFC|>1) related to AML relapse (up-regulated genes: red, down-regulated genes: green); **B-D.** Gene co-expression network analysis based on the DEGs related to AML relapse.

**Supplemental File 2: Figure S2. Identification of DEGs based on SPNS3 expression.**

SPNS3: spinster homolog 3; DEGs: differentially expressed genes

**Supplemental File 3: Figure S3. SPNS3 might participate in the S1P signaling pathway to influence cell apoptosis. A.** SPNS3 expression negatively correlated with SGPP1 expression (R=-0.322, ***P***<0.000) in the TARGET-AML database; **B.** SPNS3 expression positively correlated with SPHK2 expression. **C.** Both of the abovementioned relationships were verified in the TCGA-AML database and compared to the relationships in normal tissues samples, and the tendency became more obvious. (AML: acute myeloid leukemia; SPNS3: spinster homolog 3; S1P: sphingosine-1-phosphate; SPHK: sphingosine kinase)

**Supplemental File 4: Table S1. Six pairs of primers were used for detecting SPNS2, SPNS3, BCL2, MCL1, BAK1 and GAPDH expression.**

| Primer | Sequence |
| --- | --- |
| SPNS3-F | CTGTCTTCGTTAGCTGCCTG |
| SPNS3-R | GCTCCTGACCACAGCAAGATAC |
| SPNS2-F | ACTTTGGGGTCAAGGACCGA |
| SPNS2-R | AATCACCTTCCTGTTGAAGCG |
| BCL2-F | GGTGGGGTCATGTGTGTGG |
| BCL2-R | CGGTTCAGGTACTCAGTCATCC |
| MCL1-F | GGAGATTCCTGACCAGAACATTG |
| MCL1-R | CGACTGGGCTTTATCAAGACAT |
| BAK1-F | GTTTTCCGCAGCTACGTTTTT |
| BAK1-R | GCAGAGGTAAGGTGACCATCTC |
| GAPDH-F | AGGTCGGTGTGAACGGATTTG |
| GAPDH-R | GGGGTCGTTGATGGCAACA |

**Supplemental File 5: Table S2. Four pairs of oligonucleotides that are designed to silence human SPNS3**

| **Primer** | **Sequence** |
| --- | --- |
| SPNS3sh1-F | CCGGCGAGGAGGTACAAGAAAGTCACTCGAGTGACTTTCTTGTACCTCCTCGTTTTTTG |
| SPNS3sh1-R | AATTCAAAAAACGAGGAGGTACAAGAAAGTCACTCGAGTGACTTTCTTGTACCTCCTCG |
| SPNS3sh2-F | CCGGCCTGAATTACATGAACTGGTTCTCGAGAACCAGTTCATGTAATTCAGGTTTTTTG |
| SPNS3sh2-R | AATTCAAAAAACCTGAATTACATGAACTGGTTCTCGAGAACCAGTTCATGTAATTCAGG |
| SPNS3sh3-F | CCGGGAGGAGGTACAAGAAAGTCATCTCGAGATGACTTTCTTGTACCTCCTCTTTTTTG |
| SPNS3sh3-R | AATTCAAAAAAGAGGAGGTACAAGAAAGTCATCTCGAGATGACTTTCTTGTACCTCCTC |
| scrambled-F | CCGGCGAGTAGAGACTGATCAACATCTCGAGATGTTGATCAGTCTCTACTCGTTTTTG |
| scrambled-R | AATTCAAAAACGAGTAGAGACTGATCAACATCTCGAGATGTTGATCAGTCTCTACTCG |
