# Supplementary figures and images for: Bioinformatics-based identification of SPNS3 (Spinster homolog 3) as a prognostic biomarker of apoptosis resistance in acute myeloid leukemia
Source: Bioengineered. 2021 Oct 5;12(1):7837–48. doi: 10.1080/21655979.2021.1982303 (PMC8806827; doi:10.1080/21655979.2021.1982303)

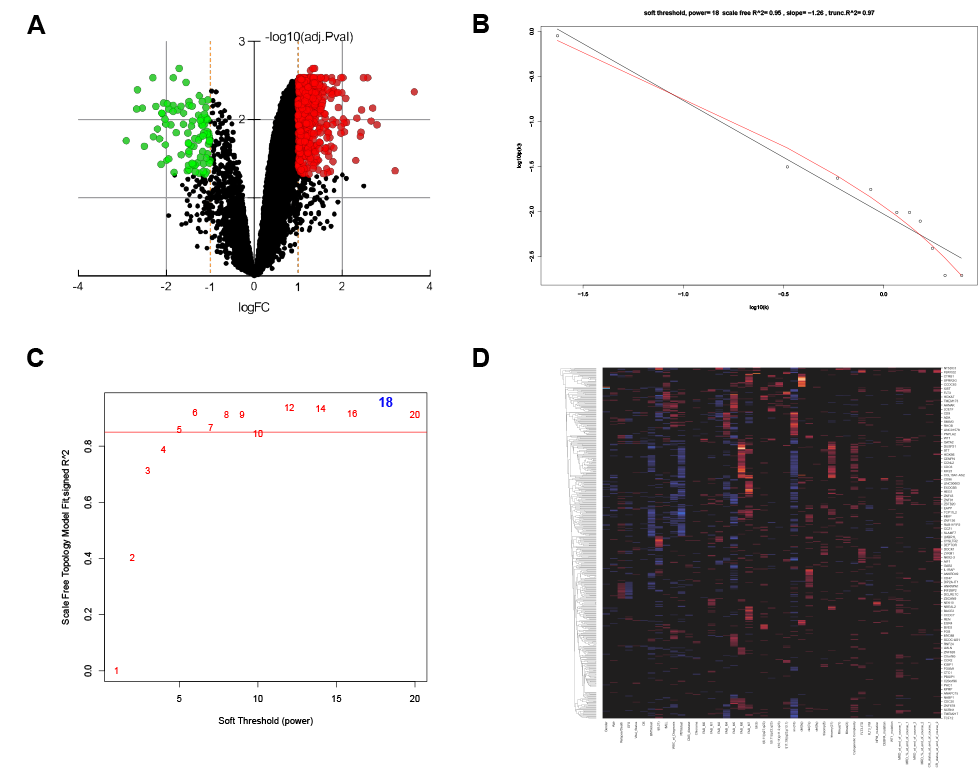

Supplement: Supplemental Material [file KBIE_A_1982303_SM6069.zip › supplementary/Supplemental File 1.tif]

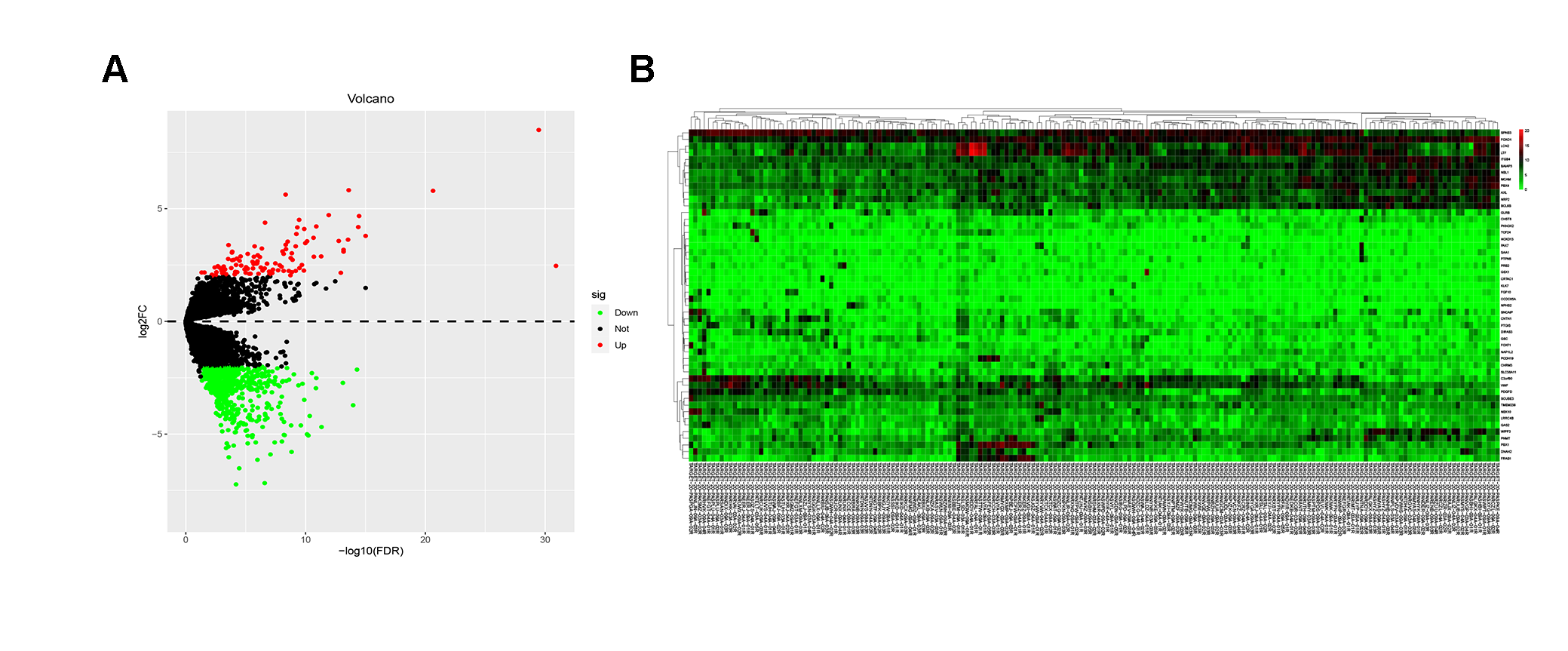

Supplement: Supplemental Material [file KBIE_A_1982303_SM6069.zip › supplementary/Supplemental File 2.tif]

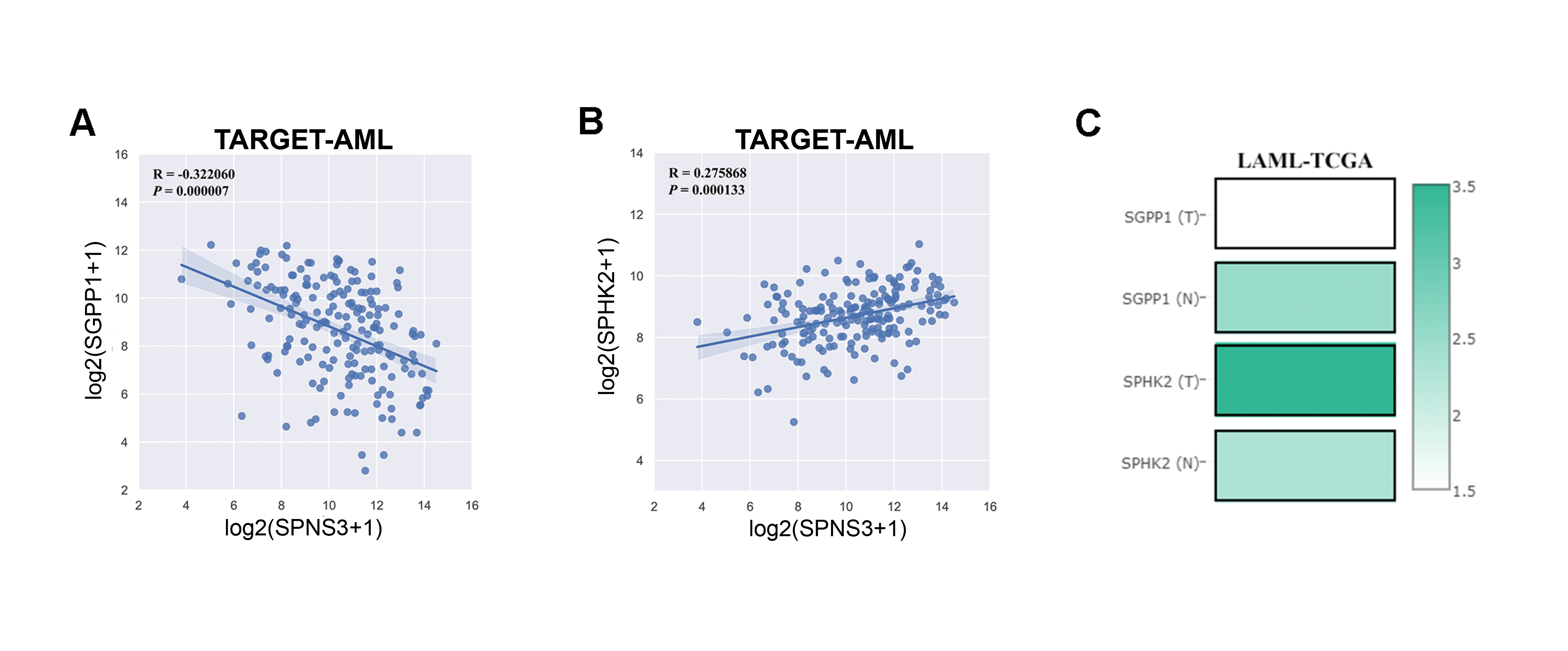

Supplement: Supplemental Material [file KBIE_A_1982303_SM6069.zip › supplementary/Supplemental File 3.tif]
